# Editorial

**Published:** 1862-01

**Authors:** 


### Editorial.

### DENTAL MEETING AT LAFAYETTE.

IN accordance with the call of the Indiana State Society, a number of dentists met at Lafayette, on Tuesday, January 14th, 1862.

As the constitution of the State Society requires a large quorum, there were not a sufficient number of its active members present to organize. Accordingly, those present organized an informal meeting, with Dr. P. G. C. Hunt in the chair, and Dr. S. B. Smith acting as Secretary.

The following members of the dental profession were present:

Drs. P. G. C. Hunt, W. W. Allport, S. B. Smith, L. Jordan, H. T. Manlove, A. M. Moore, J. S. Snoddy, Wm. H. Pifer, J. W. Fahnestock, J. Taft, and Geo. Watt.

Drs. Taft, Manlove, and Moore were appointed a committee on the order of business, who reported as follows:

I. Reading Essays.
II. Remarks on the same.
III. Periostitis—its Pathology and Treatment.
IV. Filling Teeth.
V. Mechanical Dentistry.
VI. Miscellaneous Business.

This report was adopted, and the President called for the reading of essays, which was responded to by Charles M. Wetherill, Ph. D.; M. D., of Lafayette, who was present by invitation, and read a highly interesting paper on "The Application of Chemistry to Dentistry," which evinces a thorough acquaintance with his own science, and an earnest appreciation of the wants and capabilities of ours, of which the reader can convince himself by a careful perusal of the article in the present number.

This paper gave rise to a free exchange of sentiment on the action of acids in the production of dental caries ; and it appeared to be the sentiment of all who expressed themselves that the well known varieties of dental decay are severally the result of as many different acids. We made no note of the remarks, and will not attempt to report them. Indeed, we are not making a report.

Dr. Taft read an essay on the "Wants of the Dental Profession," and another on "Dental Periostitis."

The thanks of the meeting were, on motion, returned to Drs. Wetherill and Taft, and copies of their papers were requested for publication.

In the treatment of periostitis, it appeared that most of those present rely mainly on leeches, as a means of local depletion, not, however, by any means, relying on depletion to the exclusion of other treatment.

Dr. Taft inquired why it is that cold applications sometimes relieve and sometimes increase the pain of periostitis, to which, in the absence of other remarks, Dr. Watt replied, but prefers to give his views hereafter, in a distinct paper, rather than have them partially reported here.

The subject of filling teeth was conducted in the style of conversation and illustration more than in accordance with ordinary parliamentary rule. This made it all the more pleasant and instructive to those present, but rendered it impossible for us to report it to those who were not. But we can waste no sympathy on some of them ; for they should have been there.

Some four or five, and perhaps more, of those present use the mallet, at least in a part of their operations, and regard it with favor. All of them use both soft and adhesive foil ; and from their varied remarks, in regard to different foils, it is pretty evident that our country and profession are blessed with a variety of good foilmakers. Dr. Watt uses, almost exclusively, crystallized gold (of his own manufacture.)

It has become a popular notion with the profession, that adhesive foil must be of *pure* gold ; but Dr. Taft reported that he had received a specimen of very adhesive foil from the manufacturers (Messrs. Abbey, if we are not mistaken), which, they state, contains five per cent. of silver. Some who were present thought this would be found an improvement, as the alloy would make harder plugs than pure gold.

The consideration of mechanical dentistry was informal, and, though highly instructive and interesting, is not reportable.

The "Miscellaneous" Department pretty nearly occupied the meeting, and consisted, to a considerable extent, in microscopic examinations of the dental tissues, exhibiting, in every variety of view, the structure of dentine, enamel, and cementum, normal and abnormal, both of the primary and permanent teeth. Various other textures and tissues were examined, including crystal gold, and several specimens in comparative anatomy. These specimens are all prepared with the utmost care, and are finely mounted, many of them by G. S. Allan, D. D. S., late of the Ohio College, which, added to the private cabinet of Dr. Taft, constitute a selection of specimens for the illustration of dental anatomy, physiology, and pathology, not, probably, surpassed in the world.

Another "miscellaneous" (and special, too,) item was an adjournment to the residence of Dr. A. M. Moore, where we were welcomed by his hospitable lady and a house full of as pleasant company as could well be collected, representing the various professions, and other occupations, from the killing of traitors down to the drawing of teeth.

In looking at the earnest, intelligent countenances of Gen. Reynolds and Captain M'Donald, his senior aid, our mind wandered away off to Cheat Mountain, and the doings and darings of our brave soldiers there, when our reverie was broken by a general charge, in which we joined, and found the enemy intrenched in the dining room of our liberal host ; and, as the General was our leader, of course our charge was both brilliant and successful, for that is *his way.* The enemy was surrounded, and his provisions captured. Though the Doctor's table was loaded with mountains of luxuries, none of them were *Cheat Mountains.*

Lafayette is a pretty good place, in spite of its *Artesian Well.* We are only sorry that it is so far from Xenia. W.

---

### NITRATE OF SILVER.

It is strange that men of thought, and men of education, should so persistently misunderstand the action of this salt on organic tissues. By some means it has been long ranked as the chief styptic, both by physicians and dentists; and having gained this rank, its title deed to the same has been but occasionally disputed.

We have been often exceedingly annoyed, when reading on the subject of hemorrhage, to find the author, after enumerating alum, tannin, and other styptics, add, "if these fail, apply *lunar caustic.*" Even our friend, Prof. Richardson, in his article on leeches, follows in the same strain. He says: "If from any cause, the hemorrhage should be excessive, it may be arrested by making firm pressure over the incision with a little lint; this failing, apply a piece of lint moistened with solution of alum or other convenient styptic; or touch the part with lunar caustic. If the bleeding is urgent, and not easily controlled, a solution of the persulphate of iron may be used." (Cosmos, Nov.) Now, why mention lunar caustic (nitrate of silver) at all, in this connection? Possibly because Pereira and others have done so before; but certainly not because it is reliable or useful as a styptic. It is a very active *caustic,* and its application causes pain. Would it not, then, be better to direct the persulphate at once, as it is a *styptic,* and its application produces less pain and irritation?

Many salts form combinations with the protein compounds of the blood. Nitrate of silver is one of them. It combines directly with albumen and fibrin, forming a semi-solid compound, often called a coagulum. Now, if this coagulum were permanent, the nitrate might be regarded as a styptic; and if it were, at the same time, firm in its texture, we could rely on this salt as a good styptic. But while it is not firm, it is soluble in an excess of either of its ingredients. The consequence is that when this salt is applied to arrest hemorrhage, a soft coagulum is formed, which may, and often does arrest the flow for a time; but if the hemorrhage is active or obstinate, the albumen of the blood dissolves its way through it, and nothing permanent is gained.

A genuine styptic forms, with some part of the blood, a compound, firm in its texture, and not soluble in fresh blood. And nothing is plainer than that the nitrate fails to fulfill these indications. It was introduced among the class of styptics before its action on living tissues was well understood; and that so many still class it thus, only shows how little original thought there is in the world.

Tannin, perchloride, and persulphate of iron are the most reliable styptic agents; and the application of any of them is less unpleasant than that of the nitrate. There is, therefore, no excuse for the use of *lunar caustic* in such cases. It should be left with the escharotics, where it naturally belongs.

Tannin forms a firmer compound with albuminous tissues than any other known substance; but the layer of coagulum is so thin that it is liable to be broken through by the mere force of the current. Were it not for this, it would be a far more reliable styptic than either of those mentioned in connection with it.

Many, no doubt, are ready to say that they have applied the nitrate with success. So has the sorceress applied her charms with success. The blood has ceased to flow, but "*post hoc non ergo propter hoc.*" This reminds us of a case in point:—A few years ago we were called to see an eminent physician, who had been bleeding some ten hours after the extraction of an upper molar. By direction of himself, and perhaps other medical friends, nitrate of silver, acetate of lead, sulphate of zinc, and finally sulphate of copper were perseveringly applied, accompanied with pressure, and other means. The hemorrhage was not arrested, except for a few minutes after each application, and was rapidly on the increase at the time of our visit. By removing the clots, and applying a solution of tannin, with moderate pressure, the flow was instantly checked, and did not return. The patient was astonished, and inquired why that application succeeded, when more powerful styptics failed. We replied that, in a chemical point of view, he had not used *styptics* at all,—simply astringents and escharotics, and that the application of the tannic acid was successful because tannate of albumen is insoluble in blood. "Every man to his department," was the modest reply of our patient, the now lamented Professor Cobb. W.

### THE USE OF THE BATTERY.

A friend writes to know if we still use the battery in extracting teeth. Well—no, that is ye—s,—well, to tell the truth, our battery is out of repair, or we would use it sometimes. It has lost one of its "strings," and is not sufficiently after the Paganini order to play well with *one string.* But to be serious. We don't know any thing about the battery, as used in extracting. We used it indiscriminately, in nearly two thousand cases, in some of which it appeared to do good. In very many cases the patients affirmed that they felt no pain. Some of them submitted to the extraction of half-a-dozen or more teeth without change of countenance, stating after the operation that they experienced far less pain than in ordinary extraction. Others thought it gave no relief in any way, and a few thought it did harm. Our own observation has led us to believe that, in many cases, the pain is greatly mitigated, and in others increased more or less. We let the battery go into disuse mainly because we were unable to discriminate—to tell what cases would be benefited, and what ones would not, by its use. We used an ordinary battery—nothing peculiar in its construction. W.

### OUR PHILADELPHIA CORRESPONDENT.

Some have appeared to think that, when an editor admits an article into the pages of his journal, he tacitly agrees with all its sentiments; but for him to exclude all that he can not fully subscribe to would be allowing but little latitude for a healthy difference of opinion. If we understand our "O. U. C.," in the present number, we do not agree with all he says, but will only give a single instance of disagreement. In speaking of the Philadelphia College, he says: "This Institution has now become the leading school in our profession ; the locality and the ability of its faculty fully warranting such a position."

The first clause of this sentence is evidently true; and the latter may be too. It may be that the said "locality" and "ability" combined fully warrant such a position; for Philadelphia is a good locality for such an Institution, as is evidenced by the fact that several of its medical schools flourish, while others, not so fortunately situated, languish, even with far abler faculties. But if "O. U. C." means that the "ability" of the Philadelphia Faculty warrants such a position, we take issue. Like our correspondent, we are outside of all faculties; and from *personal* acquaintance we frankly give our opinion, that in all the dental colleges of America no faculty has less "ability" than that of "the leading school." And this statement can not be interpreted into even an apparent disrespect to the faculty referred to; for no set of men in existence are more *contentedly* conscious of the truth of the statement than the members of this faculty. We rejoice in the prosperity of the Pennsylvania College; and it has reason to rejoice that, unlike its fellows, it is some distance from the war. We hope it will still prosper, and be in the future, as it has been in the past, a blessing to the profession. W.

### THE MISSISSIPPI VALLEY ASSOCIATION.

The annual meeting of the Mississippi Valley Association will be held on Wednesday, the 19th of February, at 10 o'clock A. M., at the Lecture Room of the Ohio College of Dental Surgery; at which time and place we hope to meet all the members of the profession who can at all make it convenient to attend. We have the assurance from quite a number from a distance that they will be here. From present indications we anticipate a good time. There will be many things to make it an interesting and instructive meeting. T.

### GOLD FOIL.

It is scarcely necessary to direct the attention of any one who has been in the dental profession any considerable time to Abbey & Son's Gold Foils. But there are two or three things we wish to say in regard to it. In the first place, they manufacture every variety of foil that is made—at least they include every principle. With their soft foils all dentists are familiar : they also prepare adhesive foil of a superior quality. We are using some of their No. 4 that we have never seen excelled ; it is very adhesive, and yet not stiff or harsh. This firm has always maintained a high reputation for the purity of their foils. It is the oldest gold foil manufacturing firm in the United States, and is doubtless in possession of all the facilities that are known for the production of perfect foil. We are confident no one will ever make a mistake in purchasing Abbey foil. T.

### OHIO DENTAL COLLEGE ASSOCIATION

WILL meet on Thursday, the 20th of February, at 9 o'clock, A. M. at the lecture-room of the College. It is important that there be a full meeting, as matters of great importance to the Institution will be presented for consideration. T.

### DELAYED.

THE present number of the Register is more than a month behind the proper time of issue. During this time we have frequently been asked, " Where is the Register ? why isn't it out ?" and various questions of this kind. We will endeavor to answer these queries as satisfactorily as we can. Up to about the 15th of January, we had nothing from our contributors to the original department, and we had resolved not to make up this number from selections and our own scribblings alone. We were waiting the movement of our contributors ; and yet we can scarcely find fault with them, for doubtless each one supposed others would write, though he was a little tardy. We hope in future, however, that our correspondents will send their articles as early as possible, without waiting to consult us, after they are prepared, as has been done in several instances recently. The February number will follow this within a few days, and hereafter we shall issue promptly. T.

WHAT THE "LOUISVLLLE JOURNAL" SAYS.

"WE do not believe that even in this age of cheap publications, any work can be more reasonable than the terms of the SCIENTIFIC AMERICAN at $2 per annum, with twenty-five per cent. discount for clubs of ten. It forms a yearly volume of 832 pages quarto, with an immense number of original engravings of patented machines, valuable inventions, and objects of scientific interest. There is not an industrial pursuit which does not receive a share of its attention. It contains official lists of patent claims, important statistics, practical recipes for useful domestic purposes, and has long stood, both in this country and Europe, as the highest authority in the mechanic arts and sciences. There is no publication more valuable to the farmer, the miller, the engineer, the iron founder, the mechanic, or the manufacturer. We have never opened a number without learning something we never knew before, and obtaining valuable information for the benefit of our readers. The Publishers, Messrs. MUNN & Co., of 37 Park Row, New York, have deserved the success which they have achieved. No one should visit that city without calling at their palatial establishment, which is a museum of inventive genius, collected from the entire world. If any of our friends away off in the country do not know this work, and will take our advice, they will mail $2 and become subscribers immediately, or by applying to the Publishers, they can obtain a specimen copy gratis, which will be sure to confirm the truth of our recommendation."

We fully indorse the above, and would recommend our readers to take Prentice's advice, and subscribe for the paper. A new volume commences on the first of January, and it being a valuable work of reference, containing, as it does, the only official list of patent claims published in the country, every number should be preserved. The paper is published every Saturday, by the well known patent agents, Messrs. MUNN & Co., who have conducted the paper during the past sixteen years.

In addition to furnishing specimen copies of the paper gratis, the publishers will send a pamphlet of advice to inventors, free of charge. Address, MUNN & Co., 37 Park Row.

